# Local area unemployment, individual health and workforce exit: ONS Longitudinal Study

**DOI:** 10.1093/eurpub/ckw005

**Published:** 2016-02-27

**Authors:** Emily T. Murray, Jenny Head, Nicola Shelton, Gareth Hagger-Johnson, Stephen Stansfeld, Paola Zaninotto, Mai Stafford

**Affiliations:** ^1^Department of Epidemiology and Public Health, University College London, London, UK; ^2^Administrative Data Research Centre England (ADRC-E), University College London, London, UK; ^3^Centre for Psychiatry, Wolfson Institute of Preventive Medicine, Queen Mary University of London, UK; ^4^Medical Research Council Unit for Lifelong Health and Ageing at University College London, London, UK

## Abstract

**Background:** In many developed countries, associations have been documented between higher levels of area unemployment and workforce exit, mainly for disability pension receipt. Health of individuals is assumed to be the primary driver of this relationship, but no study has examined whether health explains or modifies this relationship. **Methods:** We used data from 98 756 Office for National Statistics Longitudinal Study members who were aged 40–69 and working in 2001, to assess whether their odds of identifying as sick/disabled or retired in 2011 differed by local authority area unemployment in 2001, change in local area unemployment from 2001 to 2011 and individual reported health in 2001 (self-rated and limiting long-term illness). **Results:** Higher local area unemployment and worse self-rated health measures in 2001 were independently related to likelihood of identifying as sick-disabled or retired, compared to being in work, 10 years later, after adjusting for socio-demographic covariates. Associations for local area unemployment were stronger for likelihood of identification as sick/disabled compared to retired in 2011. Associations for changes in local area unemployment from 2001 to 2011 were only apparent for likelihood of identifying as retired. For respondents that identified as sick/disabled in 2011, effects of local area unemployment in 2001 were stronger for respondents who had better self-rated health in 2001. **Conclusions:** Strategies to retain older workers may be most effective if targeted toward areas of high unemployment. For persons in ill health, local area unemployment interventions alone will not be as efficient in reducing their exit from the workforce.

## Introduction

In many industrialised countries, population ageing has prompted governments to raise age requirements for state pension eligibility in order to reduce fiscal demands on budgets.^1^ Extending working lives can also have individual benefits,^2^ including delaying retirement to build up monetary reserves^3^^,^^4^ or reducing personal debt. However, uniform postponement of pensionable age may be inappropriate because retention of older persons in the workforce is not distributed equally across geographical areas.^5–10^

It is important to understand the contextual characteristics of areas that may lead to disparate rates of workforce exit for government planning, and creation of interventions to reduce inequalities in worker retention. In recent decades, correlations have been documented between higher levels of area unemployment and a higher prevalence of disability pension usage.^7–9^ Recently, two Finnish studies,^10–11^ using one dataset, showed that local area unemployment predicted incidence of disability pension uptake. However, to establish a causal relationship, one would want to see that changes in area unemployment, rather than single-point rates, predict workforce exit. To date, only one Icelandic study^9^ has shown that this was the case, reporting that country-wide disability pension incidence increased when the unemployment rate rose.

What none of the previous studies has done, and is important for policy and intervention-design, is to examine the role that health plays in the relationship between local area unemployment and workforce exit. Ill-health is a major predictor of both receipt of disability benefit^12^ and retirement.^5^^,^^13^^,^^14^ But higher proportions of ill persons also tend to reside in areas of high unemployment.^15^ If relationships between area unemployment and workforce exit can be explained by the distribution of ill-health across areas, then interventions should be focused on the individual. But if health modifies relationships between area unemployment and workforce exit, such as from job destruction focused first on workers struggling with job demands,^12^^,^^16^ then policy will need to be applied at the contextual and individual level.

In addition, most studies examining area-level influences on workforce exit have focused on rates of disability benefit receivership. Effects of local area unemployment on total workforce exit would be missed, or underestimated, in samples including older workers who were ill, but not ill enough to qualify for disability benefits; potentially a large percentage of older persons as most economic inactivity in the UK of the aged over 60 is mostly due to retirement.^17^

This study therefore aimed to determine in England and Wales, whether local area unemployment in 2001, and change in local area unemployment 2001–2011, were related to individuals differentially identifying as sick/disabled or retired in 2011. In addition, we assess whether these effects could be explained by individual health, or whether they differed for persons with varying health states in 2001.

## Methods

### Study participants

The Office for National Statistics Longitudinal Study (LS) is a 1% representative sample of the population of England and Wales, drawn initially from respondents to the 1971 census that had been born on one of four birthdays. New members are added to the LS if either newly born or immigrants had the same birthdays. Additional 1% samples have also been drawn from the 1981, 1991, 2001 and 2011 censuses, as well as each sample being followed up. All longitudinal data used for this study was extracted from linked 2001 and 2011 census responses. The sample for this analysis included individuals in work, aged 40–69 in 2001; chosen for their representativeness to individuals being targeted by the Extending Working Life Sector Initiative, a government programme aimed at extending employment rates for individuals aged 50+ years.^18^

### Work status variables

At both the 2001 and 2011 censuses, respondents completed questions to determine their employment status in the week preceding each census (Supplementary table S1).^19^^,^^20^ Additionally, in 2011 individuals were asked ‘Last week, were you: (tick all that apply)’: ‘retired’, ‘a student’, ‘looking after home or family’, ‘long-term sick or disabled’ or ‘none of the above’. Using these questions, a four-category variable was created to characterize an individual’s work status in 2011: (i) In work, or not in work and self-identified as (ii) sick/disabled, (iii) retired or (iv) other. As more than one non-work category could be chosen, any mention of ‘sick/disabled’ was prioritized first, followed by retired (Supplementary table S2).

### Area unemployment indicators

At both censuses, local authority of each respondent’s usual residence was noted. Staff at the Center for Longitudinal Study Information and User Support (CeLSIUS) then linked each LS member’s local authority identifier in 2001 and 2011 to local authority population-based aggregate census employment data, obtained from the UK Data Services InFuse data wizard. LS members resided in all local authorities in England and Wales in 2001 (*n* = 375) and 2011 (*n* = 383), with a median of 228 (range 2–1395) LS members per local authority in 2001.^21^^,^^22^ Two area unemployment indicators were created from this data: (i) baseline local area unemployment in 2001 (classified into tertiles)—percentage of person’s in a local authority classified as ‘unemployed’, out of the number of person’s aged 16–74 who were actively looking for work (ONS table KS09a in 2001 and KS601c in 2011) and (ii) ‘change in local area unemployment’—the percentage point difference in local authority unemployment between 2001 and 2011, classified as, ‘improvement’ (<−0.50), ‘no change’ (−0.50 to 0.50% difference), ‘minor deterioration’ (0.50–1.42%) or ‘high deterioration’ (1.43– 3.68%). The ‘deterioration’ category was split evenly into two categories as such a large percentage of the sample experienced deterioration (85.4%), determined by the available data rather than by theory.

### Health conditions

Two health indicators were assessed at the 2001 census: (i) limiting long-term illness (LLTI)—‘a long-term illness, health problem or disability which limits your daily activities or the work you can do’ and (ii) self-rated health—‘over the last 12 months would you say, your health has on the whole been: good, fairly good or not good?’^23^ Previous work has shown that both are required for accurate proxies of an individual’s health state.^24^

### Covariates

Socio-demographic indicators in 2001 were investigated as potential confounders, including: (i) age, classified into six categories of 40–44 years, 45–49 years, 50–54 years, 55–59 years, 60–64 years or 65–69 years, (ii) gender, (iii) employment status into three categories of full-time, part-time or self-employed (iv) ethnicity, classified into four categories of white, Asian, black or other/mixed and (v) housing tenure, classified into four categories of owner, mortgage, rent or other. Occupational social class was based on the Registrar General’s classification,^25^ collapsed into four categories of professional/managerial, skilled non-manual, skilled manual or partly /un-skilled.

### Statistical analysis

All socio-demographic, health and area predictors were compared across 2011 work status categories (In work, sick/disabled, retired and other) using Analysis of Variance (ANOVA) for continuous variables and the chi-square statistic for categorical variables. Multinomial logistic regression, interpreted as a multivariate binary model,^26^ was used to assess associations between each predictor and the odds of self-identifying as one of the non-work statuses, compared with remaining in work in 2011. Generalized estimating equations were used to account for correlations between persons within local authorities. The model was defined as:

$$\text{Log}\left({\text{p}}_{\text{jr}}/\text{1}-{\text{p}}_{\text{jr}}\right)\;=\;{ß}_{0\text{r}}+{\text{ X}}_{\text{ij}}{ß}_{\text{r}},\text{ r}=\text{2},\ldots ,\text{R}$$

Where r is the response category for work status in 2011 [1 = working (reference category), 2 = sick/disabled, 3 = retired, 4 = other], p_jr_ are expected response probabilities for work status, *ß*_0_r is the log odds of the binary response category for person i residing in local authority j when X_ij_ = 0, and *ß*_r_ the change in the logs odds of the binary response category with a 1-unit change in covariate X_ij_ (characteristic for person i residing in local authority j). PROC GENMOD with a ‘repeated’ statement was used to model correlations within local authorities; the subject effect was specified as an interaction of the intercept with the original subject variable, and an independent working correlation matrix.

First, we fitted two separate models, one with local area unemployment in 2001 and change in local area unemployment 2001–11 only (model 1) and the other with 2001 individual health indicators of self-rated health and LLTI only (model 2). To assess whether local area unemployment effects could be explained by the health of individuals, both local area unemployment and health indicators were fitted simultaneously (model 3). To determine whether effects of local area unemployment differed by gender or health status, *P* values were assessed for interaction terms, added to models separately for each covariate (21 tests). Lastly, model 3 was further adjusted for individual demographic characteristics of age, gender, employment status, social class, ethnicity and housing tenure (model 4). To account for differences in workforce exit by gender and age group (Supplementary table S3)—potentially due to differences in state pension ages,^27^ caring responsibilities,^27^ disability benefit receivership,^28^–an interaction term of age (in 5-year bands)*gender was also included. To investigate whether effects of changes in local area unemployment could be a consequence of work exit, analysis was run separately for individuals who did and did not move residence.

## Results

**Figure 1:**
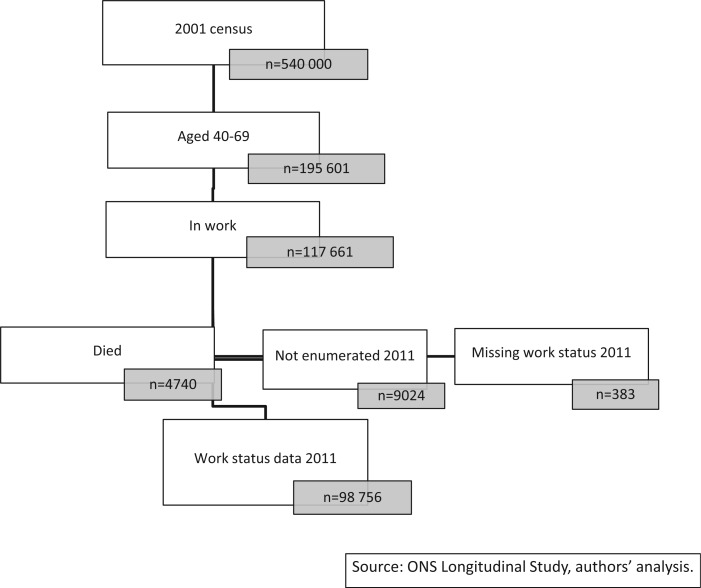
Flow of respondent's included in the analysis

Of the 117 661 LS members working in 2001 and aged 40–69, 9024 were not enumerated in 2011, 383 were missing work status in 2011, and 4740 died; resulting in a sample of 98 756 individuals (figure 1). The distributions of individual and contextual characteristics of the sample, both in 2001 and by working status in 2011, are located in table 1. Self-identified working status in 2011 was associated with all individual and contextual factors measured in 2001, though not with change in local area unemployment 2001–11 (table 1).

**Table 1:** Distribution of study participant’s characteristics 2001 by work status in 2011

	2001	Working status 2011						
		InWork	Not in Work					*P* values (diff in row percentages)
			Sick/Dis.		Retired		Other	
Total	N = 98 756 (100.0%)	64.9%	3.0%		28.5%		3.6%	
**Individual factors**								
*Socio-Demographic*								
Gender								
Males	53 510 (54.2)	67.1	3.1		26.7		3.2	<0.001
Females	45 246 (45.8)	62.3	3.0		30.7		4.1	
Age group (years)								
40–44	26 611 (27.0)	90.0	2.9		1.7		5.4	<0.001
45–49	24 144 (25.8)	82.3	4.3		7.6		5.9	
50–54	23 779 (25.4)	57.8	3.5		36.4		2.3	
55–59	15 442 (16.5)	29.6	1.5		68.5		0.5	
60–69	8780 (9.4)	21.2	1.7		75.6		0.4	
Employment status								
Full-time	59 425 (60.5)	66.6	3.1		27.1		3.3	<0.001
Part-time	21 889 (22.2)	56.2	3.3		35.9		4.6	
Self-employed	17 442 (17.8)	70.1	2.7		24.0		3.3	
Social class								
I–Prof/Managerial	40 127 (41.1)	69.0	1.8		26.5		2.8	<0.001
II–Skilled non-manual	20 700 (21.2)	62.2	2.7		31.3		3.8	
III–Skilled manual	19 148 (19.6)	65.4	4.1		26.7		3.8	
IV–Partly/un-skilled	17 574 (18.0)	58.3	5.1		31.7		4.9	
Ethnic group								
White	92 916 (94.1)	64.6	3.0		29.0		3.4	<0.001
Asian	3982 (4.0)	68.7	4.5		20.6		6.3	
Black	1162 (1.2)	73.7	3.0		18.6		4.7	
Other	696 (0.7)	72.0	3.2		19.1		5.8	
Tenure								
Owner occupier	26 227 (26.9)	49.5	2.4		45.4		2.8	<0.001
Mortgage	60 508 (62.0)	71.8	2.7		22.0		3.5	
Rent	10 257 (10.5)	63.0	6.9		24.0		6.1	
Other	630 (0.7)	69.7	4.8		21.6		4.0	
*Health*								
Limiting long-term illness								
Yes	8692 (8.8)	48.9	10.0		37.8		3.3	<0.001
No	90 064 (91.2)	66.4	2.4		27.6		3.6	
Self-reported health								
Good	70 412 (71.3)	68.3	1.9		26.5		3.4	<0.001
Fairly good	24 377 (24.7)	58.0	4.8		33.2		4.0	
Poor	3967 (4.0)	47.5	13.2		35.5		3.8	
**Contextual factors**								
% unemployment 2001								
Low (≤3.56)	32 705 (33.1)	65.8	2.2		28.8		3.1	<0.001
Middle (3.56–5.25)	32 803 (33.2)	64.9	3.0		28.5		3.6	
High (≤5.25)	32 298 (33.7)	63.9	3.9		28.2		4.0	
Percentage point change unemployment 2001–11								
Improvement ( < −0.5)	3257 (3.3)	68.5	3.1		24.7		3.7	0.5937
None (−0.5 to 0.5)	11 182 (11.3)	65.4	3.4		27.9		3.4	
Minor deterioration (0.5–1.4)	42 114 (42.6)	64.6	3.2		28.2		3.9	
High deterioration (1.4–3.7)	42 203 (42.7)	68.5	3.1		24.7		3.7	

For presentation purposes, the odds ratio for identifying as sick/disabled or retired in 2011, vs. being in work, are shown in table 2 (disabled) and table 3 (retired). Concerning gender interaction, out of 21 tests there was only one significant term, indicating that the association between high local area unemployment in 2001 and lower odds of identifying as retired in 2011 was larger in magnitude for men compared with women (*P* = 0.01). However, differences were not large and in the same direction (data available from the authors), so results are presented with genders combined.

**Table 2:** Odds ratio of reporting being sick/disabled in 2011, vs. in work, by local authority area unemployment conditions and individual health status in 2001 (*n* = 98 756)

	Model 1: Area only	Model 2: Health only	Model 3: Area and Health^a^	Model 4: + Individual Demographics^a^^,^^b^
Area unemployment indicators
*2001 only (%)*				
Low	—		–	–
Middle	1.34(1.20–1.49)		1.46(1.24–1.70)	1.41(1.20–1.66)
High	1.79(1.61–2.00)		1.92(1.65–2.25)	1.69(1.44–1.98)
*2001–11 (percentage point change)*				
Improvement ( < −0.5)	0.81(0.65–0.99)		0.83(0.66–1.03)	0.79(0.63–1.01)
None (−0.5 to 0.5)	—		—	—
Mild deterioration (0.5–1.4)	0.96(0.84–1.10)		0.95(0.84–1.08)	0.98(0.86–1.12)
High deterioration (1.4–3.7)	1.03(0.91–1.17)		1.00(0.88–1.13)	0.99(0.87–1.12)
**Health**				
*Limiting long-term illness*		2.40(2.17–2.65)	2.39(2.16–2.64)	2.58(2.34–2.86)
*Self-reported health*				
Good		—	—	—
Fairly good		2.26(2.07–2.46)	2.64(2.23–3.12)	2.49(2.10–2.95)
Poor		4.73(4.19–5.34)	5.99(4.79–7.49)	5.96(4.76–7.45)
Self-reported health*				
**Unemployment 2001**				
*Fairly good × Middle			0.78(0.62–0.97)	0.76(0.61–0.95)
*Fairly good × High			0.80(0.66–0.98)	0.76(0.57–1.01)
*Poor × Middle			0.78(0.59–1.03)	0.83(0.67–1.02)
*Poor × High			0.66(0.50–0.87)	0.65(0.49–0.86)

Source: ONS LS, authors’ analysis

^a^Includes interaction terms for area unemployment in 2001 self-rated health.

^b^Age, gender, age gender, employment status, social class, ethnicity and housing tenure

**Table 3:** Odds ratio of reporting being retired in 2011, vs. in work, by local authority area unemployment indicators and individual health status in 2001 (*n* = 98 756)

	Model 1: Area only	Model 2: Health only	Model 3: Area and Health	Model 4: + Individual Demographics^a^
**Area unemployment indicators**				
*2001only (%)*				
Low	—		—	—
Middle	1.00(0.96–1.04)		0.99(0.95–1.03)	1.09(1.03–1.16)
High	1.00(0.96–1.04)		0.98(0.93–1.02)	1.24(1.17–1.32)
*2001–11 (percentage point change)*				
Improvement ( < −0.5)	0.85(0.74–0.97)		0.85(0.74–0.97)	0.83(0.65–1.07)
Minor deterioration (0.5–1.4)	1.02(0.96–1.08)		1.01(0.95–1.07)	1.15(1.06 – 1.25)
None (−0.5 to 0.5)	—		—	—
High deterioration (1.4–3.7)	1.07(1.00–1.13)		1.06(0.99–1.13)	1.16(1.07–1.75)
				
**Health**				
*Limiting long-term illness*		1.40(1.33, 1.47)	1.40(1.33–1.47)	1.06(0.99–1.12)
*Self-reported health*				
Good		—	—	—
Fairly good		1.31(1.27, 1.35)	1.31(1.27–1.36)	1.13(1.08–1.17)
Poor		1.27(1.18, 1.36)	1.27(1.18–1.36)	1.13(1.03–1.25)

^a^Age, gender, age gender, employment status, social class, ethnicity and housing tenure.

**Figure 2:**
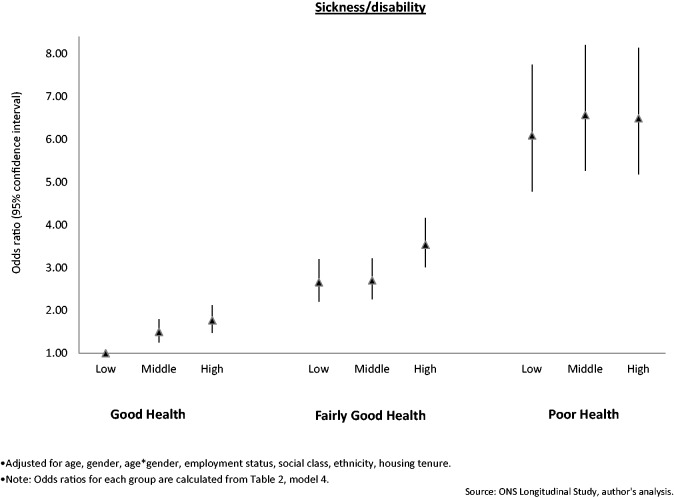
Odds ratios of reporting being sick/disabled in 2011, vs. in work, stratified by local authority area unemployment conditions and individual health status in 2001 (n = 98 756)

Table 2 shows that residence in a local area with higher unemployment in 2001 was associated with increased odds of identifying as sick/disabled in 2011, vs. being in work [high vs. low tertile: 1.79 (95% CI 1.61–2.00)] (table 2, model 1), adjusting for change in local area unemployment from 2001 to 2011. In addition, ‘improvement’ in local area unemployment from 2001 to 2011 was associated with decreased odds of identifying as sick/disabled in 2011 [0.81 (95% CI 0.65–0.99)], vs. being in work. For the health indicators only model (table 2, model 2), both an LLTI and worse self-rated health were independently related to higher odds of identifying as sick/disabled in 2011, vs. being in work. In the model including all local area unemployment and health variables (table 2, model 3), effects of local area unemployment in 2001 on identifying as sick/disabled in 2011 were strengthened by adjustment for individual health indicators. Alternatively, associations of ‘improvement’ in local area unemployment from 2001 to 2011 with decreased odds of identification as sick/disabled in 2011 were attenuated. In addition, interaction terms between local area unemployment in 2001 and self-rated health were statistically significant (middle local area unemployment 2001/fairly poor self-rated health *P* = 0.02; high/fairly poor = 0.03; middle/poor = 0.09; high/poor ≤0.01) so results are presented including these interaction terms (table 2, model 3). Adjustment for socio-demographics did not appreciably alter results (table 2, model 4). Figure 2 displays the interaction between local area unemployment in 2001 and individual self-rated health, illustrating that although poor self-rated health was consistently associated with higher odds of sickness/disability identification in 2011, the effect of local area unemployment was stronger for individuals with better self-rated health.

Factors related to identifying as retired (table 3) were different from factors predicting sick/disabled identification in 2011. Both health indicators were independently associated with higher odds of identifying as retired, vs. still being in work (table 3, model 2). Both higher local area unemployment in 2001 [high vs. low tertile: 1.24 (95% CI 1.17–1.32)] and deterioration in local area unemployment from 2001 to 2011 [minor 1.15 (95% CI 1.06–1.25), high 1.16 (1.07–1.75)] were associated with greater odds of identifying as retired in 2011, but only after adjustment for socio-demographics; particularly age group (table 3, model 4). The association between ‘improvement’ in local area unemployment and decreased odds of identifying as retired was explained by socio-demographics (table 3, models 3 and 4).

## Discussion

In this large, nationally representative LS of older adults resident in England and Wales and working in 2001, relationships were apparent between low area unemployment rates in 2001, and change from 2001 to 2011, and the odds of individuals self-identifying as sick/disabled and retired in 2011. These longitudinal associations were not explained by self-rated health or LLTI status. However, surprisingly, effects of local area unemployment in 2001 on sickness/disability identification in 2011 were smaller for individuals with worse self-rated health.

The finding that older workers living in areas with higher unemployment in 2001 were more likely to identify as sick/disabled ten years later builds on previous cross-sectional studies showing associations between area unemployment and disability pension receipt.^7–11^ For the first time, we also show this relationship for self-identified retirement as well. Associations were smaller in magnitude than those between local area unemployment and sick/disabled identification, but population effects could be larger as more persons identified as retired (24.7%) than sick/disabled (3.0%) in 2011. Why relationships between local area unemployment and retirement were only apparent after adjustment for socio-demographics is likely due to strong relationships between age and retirement, and that age distributions of high unemployment areas tend to be younger than lower unemployment areas (data not shown).

Consistent with one Icelandic study,^9^ we also found that increases in local area unemployment rates were related to higher sickness/disability, although our measure was self-identified and theirs register-based disability pension data. We expand on the Icelandic study by showing that relationships were apparent at geographies smaller than country-level and were partly explained by socioeconomic characteristics of persons who resided in local authorities where employment conditions had improved, or moved to a local authority with improved employment conditions. However, conclusions of no effect should be viewed cautiously because of the small numbers of persons who identified as sick/disabled in 2011 and experienced an improvement in local area unemployment from 2001 to 2011.

Surprisingly, deterioration in local area unemployment conditions were not related to increased odds of identification as sick/disabled. A potential explanation is that in contrast to previous recessions where older workers may have been encouraged to take a disability benefit,^29^ employers and government responses to the recent recession has been to retain workers through flexible working options, and restrict eligibility for disability benefit.^30^ We did however show that increases in area unemployment were related to an individual’s odds of identifying as retired. Tightening of eligibility for disability benefit could have pushed persons wanting to exit the workforce for health reasons to retire instead.

An unexpected finding was that effects of local area unemployment on sickness/disability appeared to be stronger for persons with better self-rated health. Stratified analysis showed that local area unemployment effects existed for all health groups, but that associations were weaker for individuals with fairly good and poor self-rated health. One potential explanation is that poor self-rated health is such a strong factor in stopping work that it overrides local area unemployment forces to some degree. However, more work is needed to replicate findings in other studies and countries.

The LS is the only British data set that includes individual employment and health data linked to population-based local area unemployment data, at multiple time points, with large numbers of respondents residing within each local authority. This allowed us to investigate whether individuals left the workforce at different rates in different economic areas; with the latter point crucial for reducing selection bias that may be present if only small numbers of individuals represent a geographic area. The major disadvantage of using the ONS dataset is that all individual data are self-identified and only available every 10 years. As a consequence, there is imprecision concerning both measurement and timing of work status and health after 2001. It would be preferable to have more frequent data points on both measures, given the importance of health ‘shocks’ in predicting retirement behaviour.^5^^,^^14^^,^^31^^,^^32^ But even though health status in 2011 was available, we chose not to include this measure due to concerns of reverse causality. We attempted to improve the accuracy of respondent’s health state by inclusion of two different health questions,^24^ however it is possible that observed associations for local area unemployment are due to residual confounding.

A further limitation is that our definition of sickness/disability is based on self-report and may not be an accurate reflection of receipt of sickness or disability benefits. In addition, as multiple work statuses in 2011 could be chosen, we chose to prioritise any mention of sickness/disability, and then retirement. There may also be small amounts of misclassification for the change in local area unemployment measure, due to changes in geographic boundaries between 2001 and 2011. The most likely outcome of this collective measurement error is that unless measurement error was higher or lower for individuals who resided in local areas of high or low unemployment, local area unemployment effects on transitions out of work are under-estimates. Lack of data between the 10-year assessments raises concerns that changes in area unemployment could be a consequence of reverse causation, as a change in work status could have prompted movement to a higher or lower unemployment area. However, this was not supported by our data, as associations did not differ for individuals who did/did not reside in the same areas in 2001 and 2011 (data not shown).

In conclusion, we provide evidence that even when individual health has been accounted for, local area unemployment rates are important predictors of stopping work and identifying as sick/disabled or retired. If these findings reflect true causal associations, strategies to retain older persons in the workforce may be most effective if targeted toward local areas with high unemployment. For persons in ill-health, additional interventions may also need to be applied, as area unemployment focused interventions will not be as effective in reducing workforce exit.
